# Effective Recycling Solutions for the Production of High-Quality PET Flakes Based on Hyperspectral Imaging and Variable Selection

**DOI:** 10.3390/jimaging7090181

**Published:** 2021-09-08

**Authors:** Paola Cucuzza, Silvia Serranti, Giuseppe Bonifazi, Giuseppe Capobianco

**Affiliations:** Department of Chemical Engineering, Materials & Environment, Sapienza, Rome University, Via Eudossiana 18, 00184 Rome, Italy; paola.cucuzza@uniroma1.it (P.C.); giuseppe.bonifazi@uniroma1.it (G.B.); giuseppe.capobianco@uniroma1.it (G.C.)

**Keywords:** PET, sensor-based sorting, plastic recycling, hyperspectral imaging, SWIR, variable selection, circular economy

## Abstract

In this study, effective solutions for polyethylene terephthalate (PET) recycling based on hyperspectral imaging (HSI) coupled with variable selection method, were developed and optimized. Hyperspectral images of post-consumer plastic flakes, composed by PET and small quantities of other polymers, considered as contaminants, were acquired in the short-wave infrared range (SWIR: 1000–2500 nm). Different combinations of preprocessing sets coupled with a variable selection method, called competitive adaptive reweighted sampling (CARS), were applied to reduce the number of spectral bands useful to detect the contaminants in the PET flow stream. Prediction models based on partial least squares-discriminant analysis (PLS-DA) for each preprocessing set, combined with CARS, were built and compared to evaluate their efficiency results. The best performance result was obtained by a PLS-DA model using multiplicative scatter correction + derivative + mean center preprocessing set and selecting only 14 wavelengths out of 240. Sensitivity and specificity values in calibration, cross-validation and prediction phases ranged from 0.986 to 0.998. HSI combined with CARS method can represent a valid tool for identification of plastic contaminants in a PET flakes stream increasing the processing speed as requested by sensor-based sorting devices working at industrial level.

## 1. Introduction

Plastics represent one of the most used materials, in daily life, in a wide range of applications, due to their peculiar characteristics and low production costs [1]. As a consequence, there has been an uncontrolled growth of large quantities of plastic waste [2], especially from packaging, still creating a series of challenges for industrialized countries at a political, economic, social, and environmental level [3]. In order to achieve circular economy and recycling targets, set by European and national legislation, to prevent the environmental impacts of plastic packaging waste, it is essential to implement efficient plastic waste recovery strategies [3,4,5]. Several actions can be taken to improve plastic recycling processes, thus allowing to bring high-quality recycled products to the market. In this context, the on-line sorting step of the mechanical recycling process plays a preeminent role in order to improve processing performance, increasing recycled plastic quality. Contaminants, i.e., other materials and other types of polymers, inside the post-consumer stream of a specific recycled polymer, can degrade the final properties of the secondary raw material [6,7,8]. A correct recognition and separation of materials in recycling plants is, thus, crucial.

Optical-based sorting of polymers is one of the key points in order to produce high-quality plastics as secondary raw materials [9,10]. Many spectroscopy- based approaches can be applied for plastic classification, including near-infrared (NIR) and short-wave infrared (SWIR) spectroscopy [10,11], Raman spectroscopy [12], and laser induced breakdown spectroscopy (LIBS) [13]. Compared with other spectroscopic techniques, NIR/SWIR spectroscopy has the advantage of rapid detection, little sample preparation and low cost [14]. NIR/SWIR spectroscopy can, thus, be profitably utilized to perform automatic plastic sorting or to implement quality control strategies in recycling plants [14]. In fact, useful information about polymers can be obtained in the NIR/SWIR range, as their molecules absorb light by overtone or combination vibrations [10].

Hyperspectral imaging (HSI), operating in the NIR or SWIR range, represents an attractive solution to characterize, identify, and classify various materials, thanks to its ability to provide information on spectral features and spatial distribution [15,16,17,18,19,20,21]. The selection of SWIR range, being characterized by a greater number of wavelengths, can allow to recognize many different polymers, even with slight spectral differences, reducing the misclassification errors [10,22,23,24,25]. HSI is based on the use of an integrated hardware and software architecture able to acquire and process data, useful to obtain spatial and spectral information of the investigated object. This information is contained in a three-dimensional dataset (i.e., two spatial dimensions and a spectral dimension), the so-called “hypercube” [9,10]. As the hyperspectral image is characterized by high-dimensional data, its spectral information is often affected by multicollinearity [26] and requires some data processing time. In fact, not all variables (i.e., wavelengths) are useful (i.e., presence of noise) or necessary (i.e., redundancy of information) to build the prediction models [27,28]. Thus, a variables selection approach could optimize classification logics of plastic waste, improving the efficiency and speed of optical sorting machines working at industrial level. The goal of variable selection is to obtain a small set of variables offering the best or at least comparable generalization or simplification capacity compared to the original set of variables [29,30]. Therefore, the variable selection can play an important role in HSI analysis before modeling in order to extract the most relevant and sensitive information. 

The proposed study was carried out to develop efficient strategies for sensor-based sorting of plastic waste in recycling plants, based on hyperspectral imaging (HSI) and variable selection approach, in particular for the production of a high-quality recycled polyethylene terephthalate (PET) flakes stream. The use of variable selection methods to reduce processing time in optical sensors-based sorting is a current goal [31,32]. In more detail, the purpose is to reduce the number of variables to be used without losing quality in the recognition during the sorting process. For this reason, the use of new techniques for variable selection can be profitable improve the quality of sorting process. 

In this study, HSI based analysis, working in the SWIR range (1000–2500 nm), was applied as a fast and non-destructive detection technique useful to obtain high predictive results. Different preprocessing strategies, among the most used in literature on NIR data [33,34,35,36,37,38,39,40,41,42,43], were evaluated to select the most efficient set or sequence, able to emphasize PET and contaminant spectral differences.

Competitive adaptive reweighted sampling (CARS) method, which performs by simulating the Darwinian “survival of the fittest” theory of evolution [44,45] was applied to eliminate the useless or irrelevant variables, and to select an optimal combination of effective wavelengths useful to recognize contaminants in a PET stream. Partial least squares discriminant analysis (PLS-DA) models were built for each preprocessing set, in order to evaluate the one showing the best efficiency to identify classes of polymers, i.e., PET and other polymers considered as a single class of contaminants.

## 2. Materials and Methods

### 2.1. Samples Overview

Plastic samples, representative of a flow-stream of PET flakes contaminated by other polymers, were collected from a recycling plant. In this scenario, the contaminants have limited and finite variability sources [46], allowing the possibility to create a representative prediction model with defined wavelengths. 

**Figure 1:**
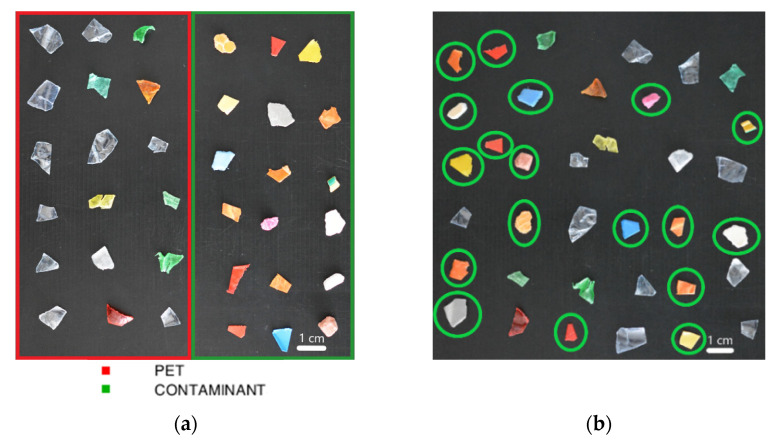
Source images of calibration dataset showing PET (red square) and contaminant (green square) flakes (**a**) and source image of the prediction dataset showing PET and contaminant flakes (the latter marked by green circles) (**b**).

Plastic flakes of PET and other polymers were selected and divided into calibration and prediction datasets for the evaluation of the PLS-DA models (Figure 1). In detail, the calibration dataset (CAL) was created from an individual image containing 36 samples divided into 18 PET and 18 contaminant flakes (Figure 1a). Principal component analysis (PCA) was used to set classes and defining the calibration set. The calibration dataset was pre-processed and cross-validated (CV) for building a PLS-DA model to detect the presence of contaminants on PET stream. The prediction image (PRED) was created from a set of plastic samples external to the model, characterized by 18 PET and 18 flakes of contaminants randomly arranged (Figure 1b).

### 2.2. Data Acquisition and Analysis

**Figure 2:**
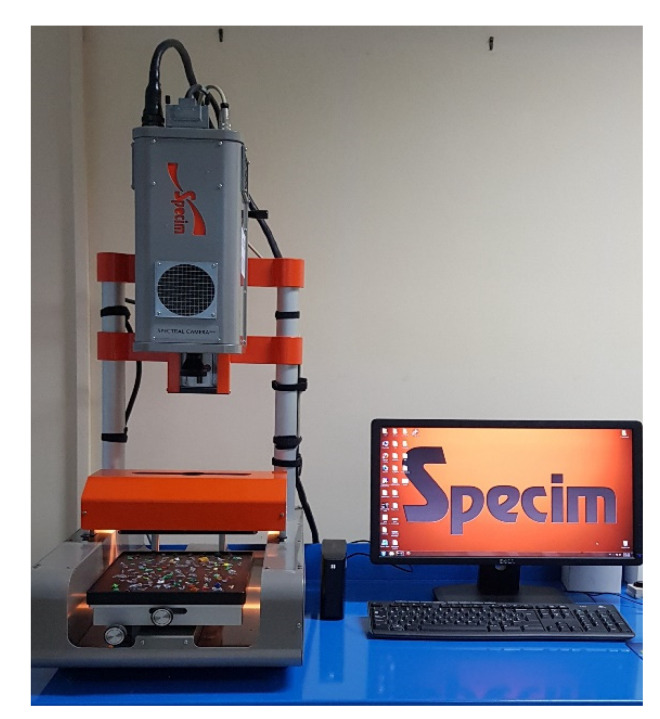
Sisuchema XL^TM^ chemical imaging workstation (Specim Ltd.).

Hyperspectral images acquisition was carried out at the Raw Materials Laboratory (RawMaLab) of the Department of Chemical Engineering, Materials and Environment of Sapienza University of Rome by the Sisuchema XL^TM^ Chemical Imaging Workstation (Specim Ltd., Oulu, Finland) (Figure 2). The HSI platform is based on a push-broom acquisition architecture, with a camera operating from 1000 to 2500 nm (SWIR range). The selected configuration of the device covers a maximum field of view of 20 cm with a pixel resolution of 625 µm. The HSI platform is equipped with a diffuse line illumination unit, consisting of quartz halogen lamps producing dual linear light, covering a spectrum range of 920 to 2514 nm, thus optimizing the imaging of various surfaces [47]. The working distance between the spectrograph lens and the sample tray plan was 30 cm. The device technical specifications are summarized in Table 1. Reflectance of hypercube was automatically set up by an internal standard reference target. A total of 240 wavelengths were collected and analyzed for each dataset. The number of pixels collected for the calibration dataset was 3420 for the PET class and 2089 for the contaminant class, while the number of pixels of the prediction dataset was 3594 for the PET class and 2836 for the contaminant class. 

PLS_toolbox (ver. 8.8 Eigenvector Research, Inc., Wenatchee, WA, USA) running in the Matlab environment (version R2020a, The Mathworks, Inc., Nertick, MA, USA) was used to analyze the acquired hyperspectral images. 

### 2.3. Data Preprocessing

Different preprocessing strategies, according to the most applied to infrared spectral data [33,34,35,36,37,38,39,40,41,42,43], including those related to plastic samples [10,15,17,48,49,50,51,52,53], were selected to build each pretreatment sequence, that is:

- Standard Normal Variate (SNV): SNV was applied to reduce the scattering effects in the spectral data and to obtain a general linearization of the relationship between signal and concentration [10,33,34,35,36,37,38,39];
- Savitzky–Golay (SG) derivative: Derivatives are a common method used to remove unimportant baseline signal from data. SG first derivative filter was applied to emphasize the spectral differences with second polynomial order and 15 points window [10,33,34,35,36,37,40];
- Multiplicative Scatter Correction (MSC): MSC is widely used for infrared data (such as SNV and derivation). MSC was useful to remove artifacts or imperfections from data, such as undesirable scatter effect [32,33,34,35,36,37];
- Smoothing: Smoothing (Savitzky–Golay routine) was used as low-pass filter (15 points) for removing high-frequency noise due to the derivation process [10,40,41,42,43];
- Detrend: Detrend was applied on spectra to remove the effects of baseline shift and curvilinearity [34,35,39];
- Mean Center (MC): Centering is one of the most common types of preprocessing, usually applied. MC has the effect to include an adjustable intercept in multivariate models [10,38,41,42,43].

### 2.4. Principal Component Analysis (PCA)

PCA is often applied for HSI data exploration, useful to provide an overview of multivariate data and to evaluate the selected preprocessing combinations [54]. PCA allows the decomposition of preprocessed spectral data into linear combinations of the original spectral data, called principal components (PCs), collecting the spectral variations in reduced set of factors. The first PCs were used to analyze the common characteristics of samples and their grouping, as the samples characterized by similar spectral signatures tend to aggregate in the score plot of the first two or three components [54].

### 2.5. Competitive Adaptive Reweighted Sampling (CARS)

CARS is an innovative and useful wavelength selection approach [44] used in NIR spectroscopy to select variables (i.e., significant wavelengths) [45]. CARS has the potential to select an optimal combination of the useful wavelengths from the full spectrum, combined with PLS regression [54]. In CARS method, regression coefficients (RC) absolute values of PLS model are used to evaluate the weight of each wavelength. Based on the importance of each wavelength, CARS sequentially selects N subsets of wavelengths by N Monte Carlo sampling run in an iterative and competitive manner. First, in each sampling run, samples are randomly selected in a fixed ratio (e.g., 80%) to build a calibration model. Then, based on RC, exponentially decreasing function (EDF) and adaptive reweighted sampling (ARS) procedures are applied to select the key wavelengths. Finally, the subset with the lowest root mean-square error of cross validation (RMSECV) is chosen.

### 2.6. Partial Least Square Discriminant Analysis (PLS-DA)

PLS-DA was used to identify predefined classes of materials (i.e., PET and contaminants), by forming discriminant functions from input variables (i.e., wavelengths) to produce a new set of transformed values useful to provide a more accurate discrimination than any single variable [55]. Venetian blind (number of data splits = 10) as cross-validation method was used, in order to evaluate the complexity of the models and to select the appropriate number of latent variables (LVs) (Figure S1). The optimal number of LVs was also determined by the smaller difference between RMSEC and RMSECV [56,57,58].

#### PLS-DA Performances

The classification performances obtained by PLS-DA models were evaluated in terms of statistical parameters: sensitivity, specificity and efficiency (Equations (1), (2), and (3)).

(1) $$\mathrm{Sensitivity}=\frac{\mathrm{True}\;\mathrm{Positive}}{\left(\mathrm{True}\;\mathrm{Positive}\;+\;\mathrm{False}\;\mathrm{Negative}\right)}$$

(2) $$\mathrm{Specificity}=\frac{\mathrm{True}\;\mathrm{Negative}}{\left(\mathrm{True}\;\mathrm{Negative}\;+\;\mathrm{False}\;\mathrm{Positive}\right)}$$

(3) $$\mathrm{Efficiency}=\sqrt{\left(\mathrm{sensitivity}\times \mathrm{specificity}\right)\;}$$

## 3. Experimental Results and Discussion

### 3.1. Average Raw Reflectance Spectra

**Figure 3:**
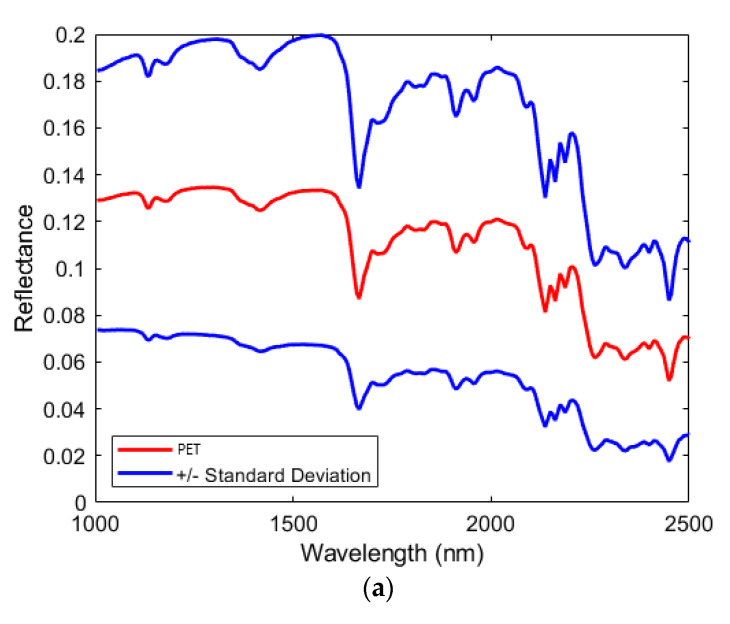

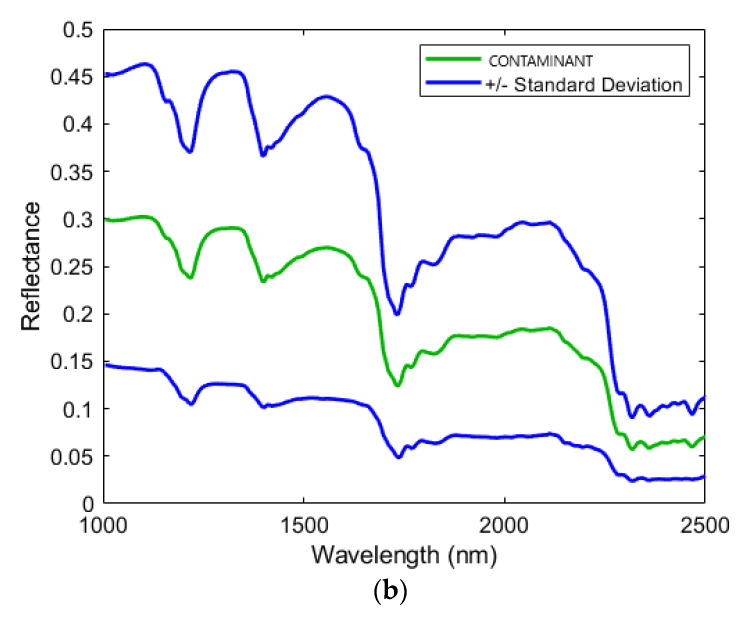
Average raw spectra and standard deviation of PET (**a**) and contaminant (**b**) classes in the SWIR range.

The average raw reflectance spectra and the standard deviation of the two classes of polymers are shown in Figure 3. PET average spectrum was characterized by absorption bands of C-H_2_ and C-H of the third harmonic region (1131 and 1182 nm), C-H of the second harmonic region (1402, 1665, 1723, 1825, 1910, and 1960 nm) and C-H stretching vibrations + C-H deformation of first combination region (2090, 2136, 2161, 2186, and 2261 nm). The average spectrum of contaminants showed a complex fingerprint due to the presence of different types of polymers, with the main absorption bands located around 1220, 1402, 1735, and 2317 nm. 

### 3.2. Preprocessing Sets and Variables Selection

In order to emphasize the spectral differences between PET and contaminants, three sets of preprocessing techniques were used, that is:

- Set 1: Detrend + Smoothing + MC;
- Set 2: SNV + MC;
- Set 3: MSC + Derivative + MC.

**Figure 4:**
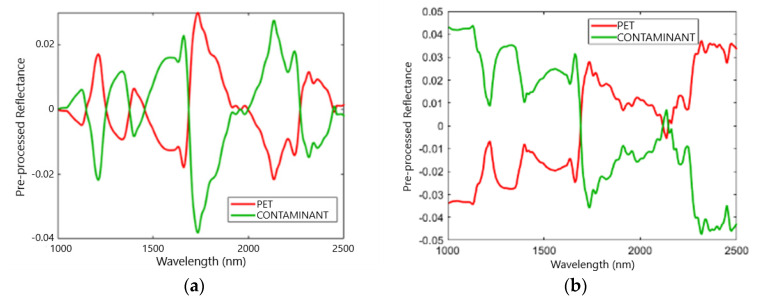

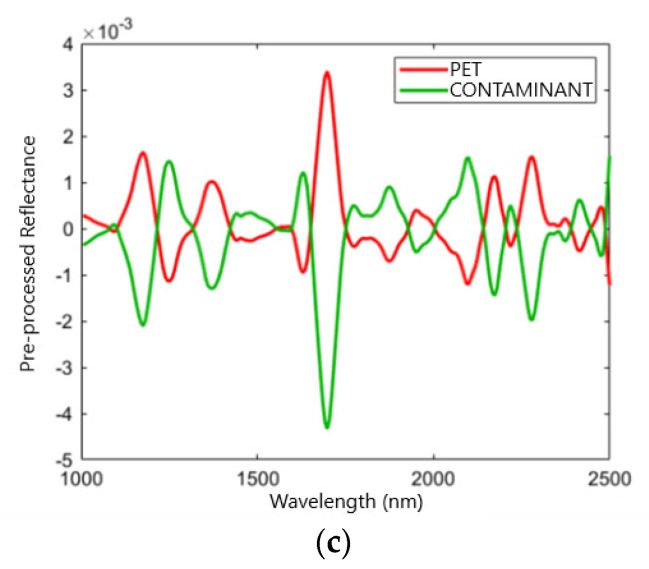
Average pre-processed spectra of PET and contaminant classes in the SWIR range adopting three sets of preprocessing techniques: Set 1: Detrend + Smoothing + MC (**a**); Set 2: SNV + MC (**b**); and Set 3: MSC + Derivative + MC (**c**).

The average reflectance spectra of the PET and contaminant classes resulting from the application of the aforementioned preprocessing sets, are shown in Figure 4.

Subsequently, for each preprocessing set, the CARS method [44] was applied, in order to reduce the number of wavelengths useful to discriminate the spectral characteristics between PET and contaminants. The selected wavelengths for each adopted preprocessing sets are shown in Table 2.

### 3.3. PCA Results of Preprocessing Set 1 (Detrend + Smoothing + MC)

**Figure 5:**
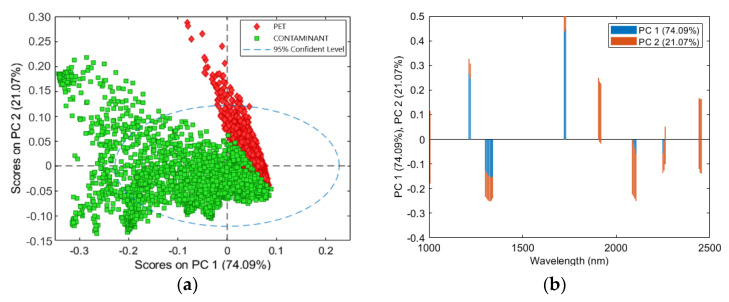
PCA results for the preprocessing Set 1 (Detrend + Smoothing + MC): PCA score plot (PC1-PC2) (**a**) and loadings plot of PC1 and PC2 related to PET and contaminant classes (**b**).

PCA score and loadings plots are shown in Figure 5. Most of the variance was captured by the first two PCs, as shown in the score plot (Figure 5a), where PC1 and PC2 explained about the 74.09% and 21.07% of the variance, respectively. The PCA score plot showed two clouds corresponding to the two analyzed classes (i.e., PET and contaminant). The cluster separation was acceptable with a low overlapping of clouds in the fourth quadrant. In more detail, the PET scores, due to the low spectral variance and high uniformity detected in PET samples, were more grouped than the contaminant scores. The variance of the contaminant was greater than the PET class, as it was influenced by the spectral combination of different polymers, as shown in the PCA score plot. The loadings plot of PC1 and PC2 was shown in Figure 5b. The main PC1 variance was given by the wavelengths around 1240 and 1720 nm for positive values, while for negative values it was mainly given by the wavelengths around 1320 and 2100 nm. PC2 was mostly influenced by wavelengths around 1730, 1910, and 2100 nm for positive values, whereas negative values were highlighted by wavelengths about 1005 and 2450 nm.

### 3.4. PCA Results of the Preprocessing Set 2 (SNV + MC)

**Figure 6:**
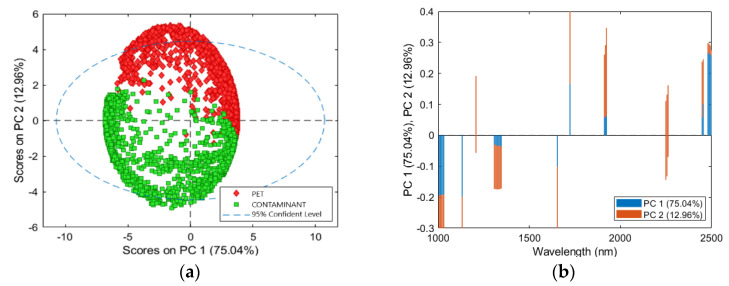
PCA results for the preprocessing Set 2 (SNV + MC): PCA score plot (PC1-PC2) (**a**) and loadings plot of PC1 and PC2 related to PET and contaminant classes (**b**).

PCA score and loadings plots are shown in Figure 6. The PCA model showed a captured variance of 95.07% with 3 PCs. The best separation between PET and contaminant clusters was allowed by PC1 vs. PC2, as shown in the PCA score plot (Figure 6a). Cluster separation was very noticeable with few overlapping pixels compared to the previous preprocessing sets. PET cluster was mainly located in the first and second quadrant, while the distribution of the contaminant scores was mainly localized in the third and fourth quadrant. Both clusters showed a similar variance distribution. Therefore, the preprocessing Set 2 (SNV + MC) approach allowed to minimize the intra-class variance, emphasizing the differences between PET and contaminant classes. The loadings plots of PC1 and PC2 are shown in Figure 6b. The PC1 variance was mainly given for positive values by the wavelengths around 1720, 2250, and 2480 nm, and for negative values by the wavelengths around 1020, 1130, and 1326 nm. PC2 was mostly marked for positive values by wavelengths around 1206, 1720, and 1920 nm, and for negative values by wavelengths around 1650 and 2255 nm.

### 3.5. PCA Results of Preprocessing Set 3 (MSC + Derivative + MC)

**Figure 7:**
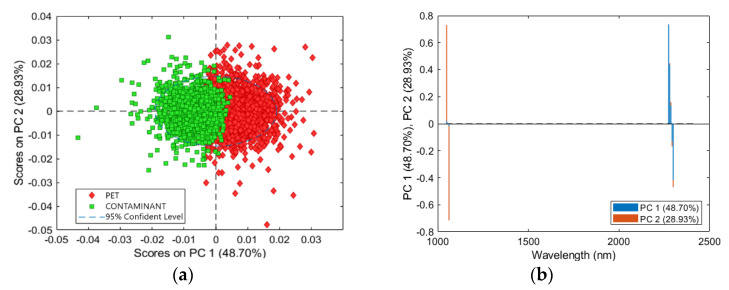
PCA results for preprocessing Set 3 (MSC + Derivative + MC): PCA score plot (PC1-PC2) (**a**) and loadings plot of PC1 and PC2 related to PET and contaminant classes (**b**).

PCA scores and loadings plots are shown in Figure 7. The PCA model showed a captured variance of 95.59% with 3 PCs. The best separation between PET and contaminants was allowed by PC1 vs. PC2. The PCA score plot showed two clusters related to PET and contaminant classes. The score plot showed a cluster separation and a low cluster overlap in the central zone (Figure 7a). PET cluster was mainly located in the first quadrant, while the class of contaminants was mainly localized in the second quadrant. Therefore, preprocessing Set 3 (MSC + Derivative + MC) allowed to minimize the intra-class variance, and to preserve the spectral differences between the two classes. The loadings plot of PC1 and PC2 is shown in Figure 7b. The PC1 variance was mainly given, for positive values, by the wavelengths around 2274 nm, and for negative values by the wavelengths around 2300 nm. PC2 was mainly influenced for positive values by wavelengths around 1050 nm, and for negative values by wavelengths around 1060 nm.

### 3.6. Classification Performances

#### 3.6.1. PLS-DA Models Constructed for a Limited Set of Spectral Variables 

**Figure 8:**
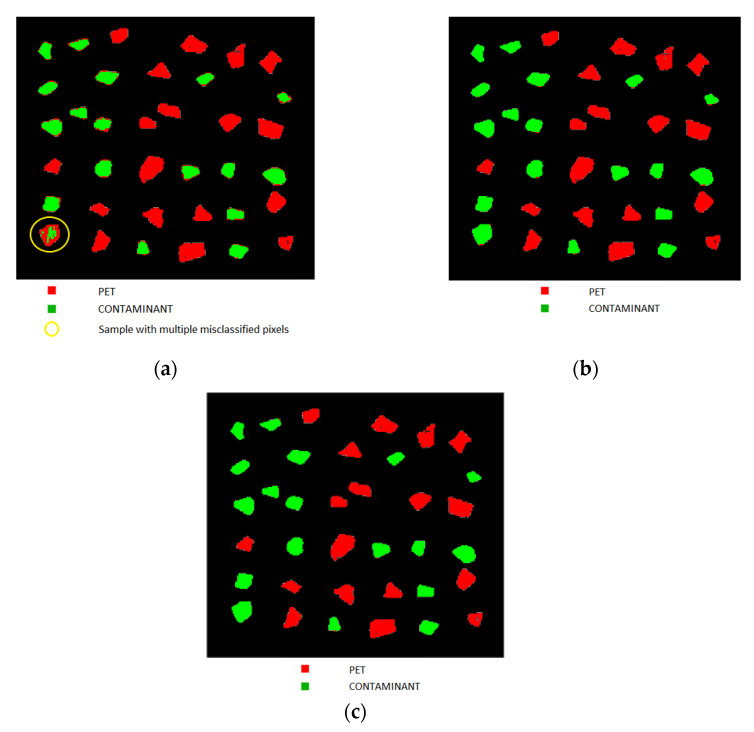
Prediction maps as resulting from the preliminary utilization of the 3 different preprocessing strategies (i.e., Set 1: Detrend + Smoothing + MC, Set 2: SNV + MC and Set 3: MSC + Derivative + MC) applied to the reduced set of wavelengths, resulting from CARS processing, and the further PLS-DA modeling. Prediction maps related to the utilized wavelengths as resulting from preprocessing Set 1: Detrend + Smoothing + MC (**a**), Set 2: SNV + MC (**b**) and Set 3: MSC + Derivative + MC (**c**).

Starting from the characteristics detected by the PCA of each preprocessing set with selected variables, a PLS-DA model was constructed. The correct number of LVs was chosen based on the smallest difference between the root mean square error for calibration (RMSEC) and cross-validation (RMSECV) values (Table 3). PLS-DA model of preprocessing Set 1 (Detrend + Smoothing + MC) showed a variance captured of 96.66% with 4 LVs; the PLS-DA model of preprocessing Set 2 (SNV + MC) showed a variance captured of 98.64% with 3 LVs and, finally, PLS-DA model of preprocessing Set 3 (MSC + Derivative + MC) showed a variance captured of 93.68% with 3 LVs. The PLS-DA models prediction results are shown in Figure 8. In general, in all models, PET and contaminant flakes samples were properly recognized using the PLS-DA model, with the presence of a few pixels not correctly classified. The only exception was related to the results achieved based on preprocessing Set 1 (Detrend + Smoothing + MC), showing a sample with multiple misclassified pixels (highlighted with a yellow circle in Figure 8) and border-effect in some flakes. The results obtained by PLS-DA models related to preprocessing Set 2 (SNV + MC) and 3 (MSC + Derivative + MC) showed a similar prediction quality, with few misclassification pixels mainly due to border-effect. However, the few pixels not correctly assigned, do not significantly affect the correct class recognition.

The classification performances obtained by the different preprocessing sets, shown in Table 4, revealed sensitivity and specificity values in calibration, cross-validation, and prediction ranging from 0.957 to 0.999. Efficiency values in prediction ranges from 0.969 to 0.991 confirming the positive quality of all PLS-DA models combined with variables selection. Based on the measured performance parameters, Set 2 (SNV + MC) and Set 3 (MSC + Derivative + MC) show a similar result in terms of specificity, sensitivity, and efficiency. However, Set 3 (MSC + Derivative + MC) is better because it uses a smaller number of wavelengths (14 for Set 3 vs. 29 for Set 2). Therefore, the best PLS-DA model was the one obtained starting from preprocessing Set 3 (MSC + Derivative + MC).

#### 3.6.2. Comparison of Full Spectrum and Reduced Wavelength PLS-DA with Preprocessing Set 3 (MSC + Derivative + MC)

**Figure 9:**
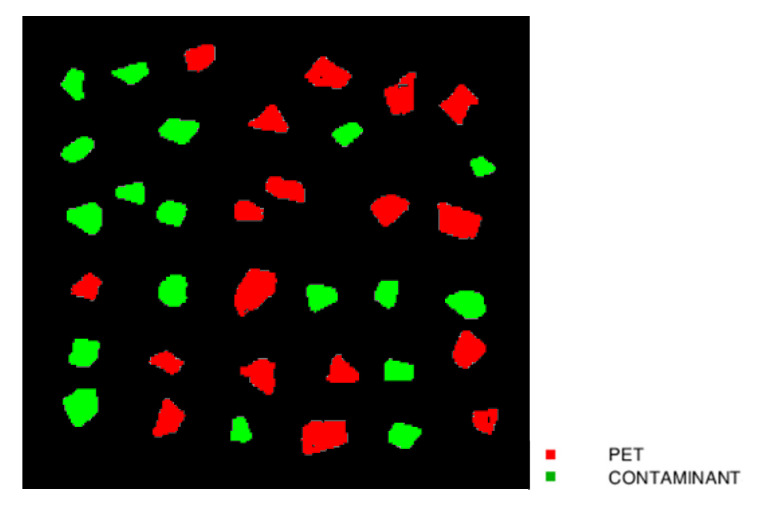
Prediction maps for the full spectrum PLS-DA model of preprocessing Set 3 (MSC + Derivative + MC).

Finally, the performances of full spectrum PLS-DA using preprocessing Set 3 (MSC + Derivative + MC) were compared with those obtained in variables selection mode with the same preprocessing set. In details, the full spectrum PLS-DA model showed a captured variance of 99.39% with 5 LVs. The LVs number was chosen based on the smallest difference between the RMSEC and RMSECV values (Table 5). Full spectrum PLS-DA prediction results are shown in Figure 9. In particular, PET and contaminant classes were well predicted, with sensitivity and specificity values in calibration, cross-validation and prediction phases and efficiency (Table 6) ranging from 0.986 to 1.000 for both classes. 

The comparison of the prediction results based on the PLS-DA-Set 3 (MSC + Derivative + MC) applied to the full spectrum hypercubes (Figure 9) and to the 14 selected wavelengths (Figure 8c) showed as they are similar. In detail, analyzing the sensitivity, specificity and efficiency values of the two models, it can be noticed a slight increase in misclassified pixel/spectra in the PLS-DA in variable selection model. However, the misclassified pixels were mainly located along the boundary of the samples, not affecting the correct attribution of the class. 

## 4. Economic and Environmental Impact

The systematic implementation of the HSI detection and classification-based logic could have important effects both at commercial-industrial and at economic-environmental level. The proposed approach can produce not only a better separation efficiency, but also a product of better quality. The fulfilment of these two goals generates social, economic, and environmental benefits [59]. In fact, in an economic viability context, a stronger and widespread PET recycling sector generates employment and contributes to reduce the volume of municipal solid waste [60]. In addition, high-quality recycled PET contributes to reduce the consumption of energy and non-renewable raw materials, [61], according to the sustainable development goals (SDGs) of UN Agenda 2030, and in particular to SDG 12, and to the principles of circular economy. 

## 5. Conclusions

The application of HSI in the SWIR region was investigated to evaluate the feasibility of a rapid and non-destructive method for the identification of plastic contaminants in a recycled PET flakes stream, producing a high-quality secondary raw material. CARS was tested as variable selection method after the application of three different preprocessing sequences to identify the best combination for the recognition of contaminants in PET stream with a limited number of wavelengths. The results of the variable selection obtained by CARS were evaluated by a PLS-DA model for each set of selected wavelengths. The best prediction results in calibration and cross-validation were provided by the combination of CARS and the preprocessing Set 3 (MSC + Derivative + MC), reducing the spectral dataset from 240 to 14 wavelengths. In addition, a comparison was made between the performances of the full spectrum PLS-DA model using preprocessing Set 3 (MSC + Derivative + MC) and those obtained in variable selection mode with the same preprocessing set. The results demonstrated that the correctness of the classification was similar, further highlighting the possibility to identify plastic contaminants in the recycled PET flakes stream using a limited number of key wavelengths, useful for online sorting applications. 

The current study supplied an effective procedure for variable selection from hyperspectral images, reducing data redundancy and obtaining a prediction efficiency close to that obtained by the full spectrum PLS-DA model. The obtained results enable the possibility to build a multispectral detection system based on filters analyzing selected spectral regions, with a significant reduction in costs compared to a conventional full spectrum hyperspectral camera and ensuring a high quality of recycled PET stream. 

## Figures and Tables

**Table 1:** SISUCHEMA XL^TM^ details (SWIR range).

Optical Characteristics		
Spectrograph	Imspector N25E	
Spectral Range	1000–2500 nm ±	
Spectral resolution	10 nm (30 µm slit)	
Spectral sampling/pixel	6.3 nm	
Spatial resolution	Rms spot radius <15 µm (320)	
Aberrations	Insignificant astigmatism, smile or keystone <5 µm	
Numerical aperture	F/2.0	
Slit width options	30 µm (50 or 80 µm optional)	
Effective slit length	9.6 mm	
Total efficiency (typical)	>50%, independent of polarization	
Stray ligth	<0.5% (halogen lamp, 1400 nm notch filter)	
Field of view (mm)	15 mm lens	
	200	
Pixel dimension (mm)	x	0.625
	y	(y dimension in mm × 0.03)/9.6
Scanning speed (mm/s)	72.50	
Scanning rate	100 hyperspectral line images/s (max), corresponding to −60 mm/s with 600 micron pixel	
**Electrical Characteristics**		
Camera	MCT camera	
Pixels in full frame	320 (spatial) × 256 (spectral)	
Active pixels	320 (spatial) × 240 (spectral)	
Pixel size on sample	Scalable from 30 to 300 µm	
Cooling	4-stage Peltier for detector array, additional Peltier for active cooling of the detector package	
Camera output	14-bit LVDS	
Signal to noise ratio	800:1 (at max signal level)	
Frame grabber	National Instruments PCL-1422	

**Table 2:** Different preprocessing sets and corresponding selected wavelengths, using CARS method.

Set	Preprocessing	Selected Wavelengths (nm)	Number of Wavelengths
1	Detrend + Smoothing + MC	1000, 1018, 1024, 1030, 1308, 1314, 1320, 1327, 1333, 1339, 1723, 1729, 1905, 1911, 1917, 2086, 2092, 2099, 2105, 2249, 2255, 2261, 2442, 2448 and 2454	25
2	SNV + MC	1000, 1018, 1024, 1030, 1131, 1207, 1308, 1314, 1320, 1327, 1333, 1339, 1346, 1346, 1654, 1723, 1911, 1917, 1923, 2249, 2255, 2261, 2448, 2454, 2479, 2486, 2492, 2498 and 2500	29
3	MSC + Derivative + MC	1049, 1055, 1062, 1119, 1291, 2217, 2224, 2274, 2280, 2286, 2292, 2299, 2411 and 2417	14

**Table 3:** Root mean square error for calibration (RMSEC) and cross-validation (RMSECV) for the three PLS-DA models constructed for preprocessing Set 1, 2, and 3.

Preprocessing Set	Classes	RMSEC	RMSECV	LVs Number
Set 1 (Detrend + Smoothing + MC)	PET	0.247965	0.248336	4
	Contaminant	0.247965	0.248336	
Set 2 (SNV + MC)	PET	0.237705	0.237803	3
	Contaminant	0.237705	0.237803	
Set 3 (MSC + Derivative + MC)	PET	0.126412	0.126612	3
	Contaminant	0.126412	0.126612	

**Table 4:** PLS-DA classification performances in variables selection mode, for calibration (CAL), cross-validation (CV) and prediction (PRED) phases.

PLS-DA Model		Classes	Sensitivity	Specificity	Efficiency (PRED)
**Set 1** Detrend + Smoothing + MC (4 LVs)	**CAL**	PET	0.974	0.989	0.969
		Contaminant	0.989	0.974	
	**CV**	PET	0.974	0.988	
		Contaminant	0.988	0.974	
	**PRED**	PET	0.983	0.957	
		Contaminant	0.957	0.983	
**Set 2** SNV + MC (3 LVs)	**CAL**	PET	0.992	0.999	0.987
		Contaminant	0.999	0.992	
	**CV**	PET	0.992	0.999	
		Contaminant	0.999	0.992	
	**PRED**	PET	0.995	0.979	
		Contaminant	0.979	0.995	
**Set 3** MSC + Derivative + MC (3 LVs)	**CAL**	PET	0.986	0.998	0.991
		Contaminant	0.998	0.986	
	**CV**	PET	0.986	0.998	
		Contaminant	0.998	0.986	
	**PRED**	PET	0.994	0.988	
		Contaminant	0.988	0.994	

**Table 5:** Root mean square error for calibration (RMSEC) and cross-validation (RMSECV) for the full spectrum PLS-DA model using preprocessing Set 3 (MSC + Derivative + MC).

Preprocessing Set	Classes	RMSEC	RMSECV	LVs Number
Set 3 (MSC + Derivative + MC)	PET	0.105549	0.105695	5
	Contaminant	0.105549	0.105695	

**Table 6:** Full spectrum PLS-DA (Set 3: MSC + Derivative + MC) classification performances for calibration (CAL), cross-validation (CV), and prediction (PRED) phases.

PLS-DA Model		Classes	Sensitivity	Specificity	Efficiency (PRED)
**Full spectrum** **PLS-DA** (Set 3: MSC + Derivative + MC)	**CAL**	PET	0.986	0.998	1.000
		Contaminant	0.998	0.986	
	**CV**	PET	0.986	0.998	
		Contaminant	0.998	0.986	
	**PRED**	PET	1.000	1.000	
		Contaminant	1.000	1.000	

## Supplementary Materials

The following is available online at https://www.mdpi.com/article/10.3390/jimaging7090181/s1, Figure S1: image of cross-validation.
