# Supplementary material for: Effective Recycling Solutions for the Production of High-Quality PET Flakes Based on Hyperspectral Imaging and Variable Selection
Source: J Imaging. 2021 Sep 8;7(9):181. doi: 10.3390/jimaging7090181 (PMC8471278; doi:10.3390/jimaging7090181)
Supplement: Supplementary file 1 [file jimaging-07-00181-s001.zip › jimaging-1341575-supplementary.pdf]

Journal of
Imaging

SUPPLEMENTARY MATERIALS

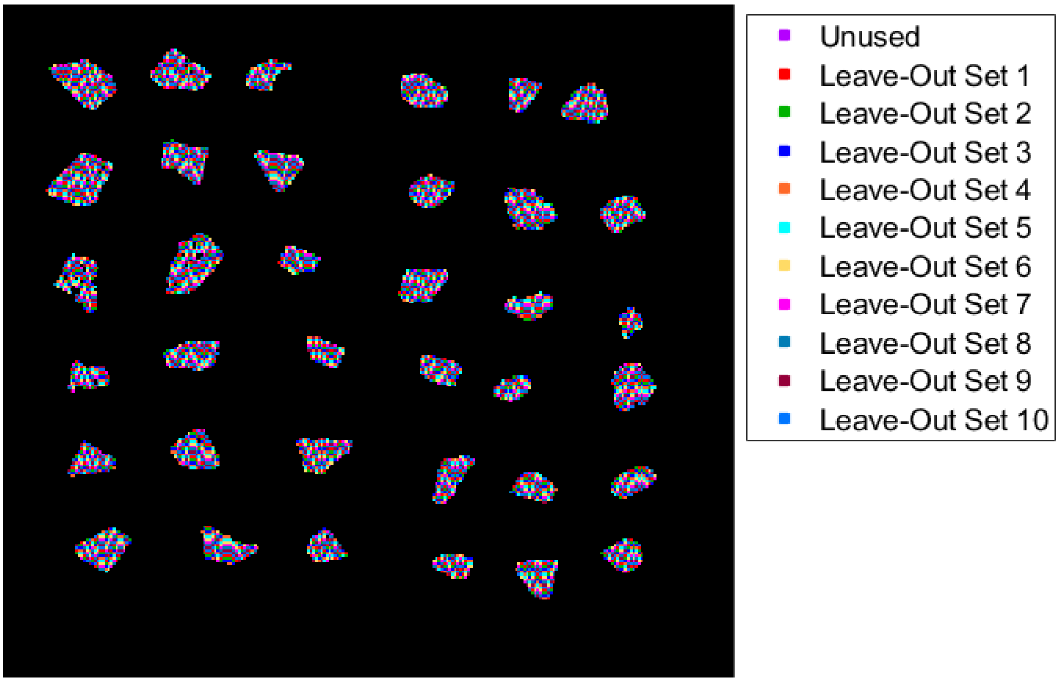

Figure S1. Venetian blind (number of data splits= 10) as cross-validation method on calibration dataset.
